# Gender Differences in Social Cognition and Their Association With Functioning in Individuals With Non‐Affective Psychosis

**DOI:** 10.1002/jclp.70052

**Published:** 2025-10-14

**Authors:** Anna‐Lena Bröcker, Helen Sauter, Dorothea von Haebler, Christiane Montag, Sandra Anna Just

**Affiliations:** ^1^ Department of Psychiatry and Neurosciences Campus Charité Mitte, Charité – Universitätsmedizin Berlin, Corporate Member of Freie Universität Berlin and Humboldt Universität zu Berlin Berlin Germany; ^2^ International Psychoanalytic University Berlin Berlin Germany; ^3^ Department of Clinical Medicine UiT – The Artic University of Norway Tromsø Norway

**Keywords:** gender, psychosis, psychosocial functioning, schizophrenia, social cognition

## Abstract

**Objectives:**

Previous studies in non‐affective psychosis (NAP) consistently found that patients' functioning is associated with social cognition and gender, with higher functioning in female patients. This study investigated the impact of social cognition on the relationship between gender and functioning, and examined psychometric properties of the Narrative Emotions Task (NET) as a measure of social cognition with high ecological validity.

**Design and Methods:**

*N* = 95 outpatients with NAP were assessed regarding functioning, social cognition, and psychopathology. Correlations were computed and a potential indirect effect of gender on functioning, mediated by social cognition, was examined.

**Results:**

Results showed a significant positive correlation between social cognition and functioning, with female patients exhibiting higher functioning. There were no gender differences in the total score of the NET, and the indirect effect of social cognition through gender on functioning could not be confirmed. Gender differences in social cognition were only evident for the NET emotion perception index. Moreover, there was some evidence of a potentially mediating effect of emotion perception concerning the relationship between gender and functioning, but this effect was not significant when covariates were added to the analysis. The NET showed high internal consistency and was correlated with an established instrument of metacognition (Metacognition Assessment Scale‐Abbreviated).

**Conclusions:**

The findings equally broaden the picture and highlight the need for further investigation into gender differences in NAP, their underlying mechanisms, and their impact on functioning. The NET appears to be a feasible measure for assessing social cognition, going beyond laboratory tasks.

## 1 Introduction

Gender^1^ has been shown to be a relevant category in schizophrenia and schizo‐affective disorders (non‐affective psychosis, short: NAP), regarding clinical, psychopathological, and (socio)cognitive aspects. Female patients on average have a later age of onset as well as a lower prevalence of comorbid substance abuse. They are more often married, have a higher degree of education and overall higher psychosocial functioning as compared to male patients (Giordano et al. 2021; Zorrilla et al. 2015; Bucci et al. 2023). Women with NAP tend to show more affective symptoms and less negative symptoms while findings for positive symptoms are inconsistent (ibid.). Regarding gender differences in social cognition, results for these diagnoses are mixed. While some studies concluded that it might be more impaired in male than in female patients (Abu‐Akel and Bo 2013; Caqueo‐Urízar et al. 2018; Pérez‐Garza et al. 2016; Zhang et al. 2017), others did not find any gender‐specific differences (Kohler et al. 2010; Navarra‐Ventura et al. 2018). Further investigation into this relationship is warranted as deficits in social cognition contribute to social dysfunction in people with NAP (Fett et al. 2011), which in turn leads to social isolation, impaired quality of life, and occupational functioning (Agerbo et al. 2004; Dziwota et al. 2018; Patel et al. 2014). In this study, we specifically aim to understand the effect of social cognition on gender differences in psychosocial functioning.

Research on social cognition dates back to the early 1970s (Green et al. 2008; Penn et al. 2008) and comprises psychological processes that enable social interaction, such as perceiving, interpreting, processing, and regulating social stimuli (Madeira et al. 2016; Pinkham et al. 2014; Wójciak et al. 2021). There is no consensus on the definition of social cognition nor on clearly delineated subdomains. Therefore, the Social Cognition Psychometric Evolution study (SCOPE; Pinkham et al. 2014) was conducted with the goal of reaching expert consensus on the major domains of social cognition, particularly for research in NAP. Experts on social cognition identified four categories of processes, partially in line with other definitions of social cognition (Addington et al. 2006; Green et al. 2008, 2015): (1) emotion processing: the perception and use of increasingly complex emotions, (2) social perception: the decoding and interpretation of social cues with the help of social rules and goals, (3) theory of mind: the ability to attribute thoughts, emotions and intentions to others, (4) attributional style: the way people typically infer causes of social events or interactions (internal, external, situational) (Pinkham et al. 2014).

Over the last 20 years, a large body of research on NAP and social cognition has emerged, but only a few studies have explicitly examined gender differences (Bucci et al. 2023). Furthermore, to our knowledge, a potentially mediating role of social cognition in explaining gender differences in functioning has not been investigated yet. Concerning gender differences in subdomains of social cognition among patients with NAP, two meta‐analyses could not find a significant influence of gender. Kohler et al. (2010) analyzed 81 studies regarding facial emotion perception and did not find gender differences. Savla et al. (2013) analyzed 112 studies and reported that variance in group differences between patients with schizophrenia and healthy control subjects in emotion perception/processing (*n* = 74; Hedge's g = 0.89), ToM (*n* = 50; Hedge's g = 0.96) and social perception (*n* = 12; Hedge's g = 1.04) was not explained by gender. However, more recent studies have found significant gender differences between male and female patients with NAP using specific measures—the self‐report measure GEOPTE Scale of Social Cognition for Psychosis (Caqueo‐Urízar et al. 2018), assessing subjective social‐cognitive deficits of patients with psychosis, the Managing Emotions subscale of the Mayer‐Salvey‐Caruso Emotional Intelligence Test, asking participants to rate the effectiveness of emotion regulation strategies (Pérez‐Garza et al. 2016; Zhang et al. 2017), and the Metacognition Assessment Scale‐Abbreviated (MAS‐A; Lysaker et al. 2005), measuring metacognition (Abu‐Akel and Bo 2013). In these studies, female patients showed significantly higher scores than male patients.

Mixed results pose the question whether gender is associated with specific aspects of social cognition and whether the reported gender differences in NAP can also be detected using other instruments. Since most of the published results come from laboratory settings, their ecological validity is limited. Measures like the MAS‐A and Narrative of Emotions Task (NET; Buck 2013) aim to achieve a higher ecological validity by rating social cognition based on patients' free and emotionally relevant narratives. Therefore, they were used in this study. Furthermore, there is need for understanding whether gender differences in functioning can be explained by gender differences in social cognition. Mediation analysis can help identify the mechanism underlying the relationship between gender and functioning, allowing us to quantify how much of the gender differences in functioning can be attributed to differences in social cognition. If social cognition is a key factor, then targeted interventions to improve social cognitive skills might help improve functioning, especially in male patients who appear to have more significant deficits in social cognition than women.

**Figure 1:**
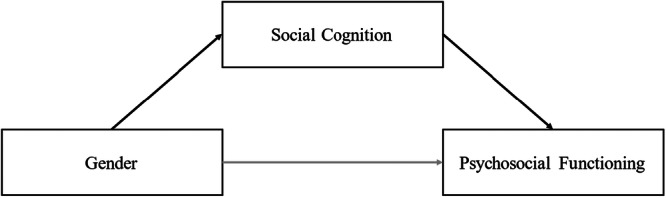
Hypothesized mediation of social cognition on the relationship between gender and psychosocial functioning.

The aim of the present study was therefore to investigate the relationship between gender, social cognition measured with the NET, and psychosocial functioning. It was hypothesized that (1) women with NAP show better psychosocial functioning than men, that (2) social cognition and psychosocial functioning show a significant positive correlation with each other, and that (3) social cognition is higher in female patients than in male patients with NAP. Ultimately, it was hypothesized that (4) social cognition mediates the relationship between gender and psychosocial functioning (see Figure 1). The study also intended to pre‐validate the German version of the NET by examining its relationship with the German version of the Metacognition Assessment Scale‐Abbreviated (MAS‐A‐G; Bröcker et al. 2017). The explorative hypothesis was that (5) the results of these two measures, which are both based on ratings of free narratives and capture overlapping domains of social cognition, are positively associated with each other.

## 2 Methods

### 2.1 Participants

The sample is a sub‐sample of the “Modified Psychodynamic Psychotherapy for Patients with Schizophrenia – a Randomized‐Controlled Efficacy Study” (MPP‐S; clinical trials ID: NCT02576613; ethics approval number: EA1/200/15). Recruiting took place from 2015 to 2018 in the outpatient sector of Berlin and Brandenburg (Germany). All patients provided informed written consent to participate in the study. Diagnosis of schizophrenia or schizo‐affective disorder was confirmed by a trained clinician according to the Diagnostic and Statistical Manual of Mental Disorders (DSM‐IV‐TR; American Psychiatric Association, 2000). Exclusion criteria included organic brain diseases or other relevant somatic diseases, active substance dependence, acute suicidality, and insufficient proficiency in German.

Interviews for measuring social cognition were available for *n* = 95 participants (37 female, 58 male) out of the overall 130 outpatients of the MPP‐S study, aged between 19 and 63 years (*M* = 38.79; SD = 10.69), who met the diagnostic criteria for either schizophrenia (*n* = 70, 73.68%) or schizo‐affective disorder (*n* = 25, 38.95%). Characteristics of illness, neuropsychological data and current medication are presented in Table 1.

**Table 1:** Characteristics of the sample (*N* ^xy^ = 95).

	Female (*n* = 37)	Male (*n* = 58)	Statistics	*p*‐value
Age (years)	40.35 (11.94)^a^	37.79 (9.79)	*t* = −1.14^d^	0.257
	19–63^b^	22–62		
Verbal IQ	105.97 (13.50)	106.24 (11.66)	*t* = 0.10	0.919
AVLT 1‐5	9.61 (2.24)	8.47 (2.58)	*t* = −2.18	0.032*
Education (years)	15.59 (3.28)	14.91 (3.30)	*t* = −1.0	0.322
Employment Status			*Χ²* = 0.24^e^	0.887
Employed	14 (37.84%)^c^	22 (37.93%)		
Unemployed	13 (35.14%)	18 (31.0%)		
(Disability) pension	10 (27.03%)	18 (31.0%)		
Duration of illness (years)	13.43 (9.45)	13.92 (10.00)	*t* = 0.24	0.812
Main diagnosis			Fisher's exact^f^	0.056
Schizophrenia (F20.0)	23 (62.16%)	47 (81.03%)		
Schizo‐affective (F25.0)	14 (37.84%)	11 (18.97%)		
Antipsychotic medication			Fisher's exact	1.00
yes	32 (86.49%)	48 (82.76%)		
no	4 (13.51%)	6 (17.24%)		
SAPS sum score	11.28 (14.78)	18.55 (15.81)	*t* = 2.20	0.030*
SANS sum score	21.2 (16.15)	26.5 (17.56)	*t* = 1.44	0.154
CDSS‐G sum score	6.11 (5.67)	5.79 (4.45)	*t* = −0.292	0.772

*Note:* Verbal IQ = Wortschatztest WST (*N* = 94); Memory & Learning = AVLT 1‐5; Employed = sheltered employment, student, in professional training; Unemployed = sick leave, seeking employment; SAPS and SANS = Scale for the Assessment of Positive and Negative Symptoms; CDSS‐G = German Calgary Depression Scale for Schizophrenia.

^a^ Mean (standard deviation).

^b^ Range.

^c^ Frequency (Percent); Group comparisons between female and male patients.

^d^ *t*‐test for independent samples.

^e^ *Χ²*‐test, ^f^Fisher's exact test.

* two‐tailed *p* < 0.05.

### 2.2 Measures

Social cognition was assessed using the *Narrative of Emotion Task* (NET; Buck 2013; Buck et al. 2014). In this task, subjects are asked to define an emotion, describe a situation in which they felt that emotion, and explain why that specific event caused that emotion (see Appendix A). The NET originally includes questions about ten emotions, while we focused on four (fear, happiness, sadness, anger) for economic reasons. Each out of four stories was rated on eight subscales from zero to three, with higher scores indicating better performance: (1) definition of emotion (“is the emotion correctly defined?”), (2) presence of narrative (“is the account given in a narrative form?”), (3) contextual appropriateness (“is the narrative normative to the target emotion?”), (4) causal inference (“is it explained why the event elicited the target emotion?”), (5) clarity of meaning (“is the content of the answer clear?”), (6) clarity of grammar (“is the grammar of the response correct?”), (7) elaboration (“how detailed is the response?”), (8) sociality (“are other people involved in the account?”).

Scores in subscales were summed up across the four emotions and three indices of social cognition were built (see Buck 2013): The scores of “definition of emotion” and “contextual appropriateness” were combined into an *emotion perception index*; “presence of narrative” and “elaboration” were summed up into a *theory of mind index*; “causal inference” represented the *attribution style index*. Considering the domains of social cognition defined by Pinkham et al. (2014), emotion processing is represented by the emotion perception index, ToM by the ToM index, and attributional style by the attribution style index, whereas social perception is not accounted for.

The total social cognition score of the NET was calculated by summing up all subscales except for sociality. Thereby, emotion perception index and theory of mind ranged from 0 to 24, attribution style index from 0 to 12, and the total score from 0 to 84. The overall internal consistency of the NET total score in this study was good (α = 0.84), according to Cronbach (1951). However, regarding the social indices and the sociality subscale, it was not acceptable: theory of mind (α = 0.68), emotion perception (α = 0.46), attribution style (α = 0.51), and sociality (α = 0.52).

The *Metacognition Assessment Scale‐Abbreviated* (MAS‐A; Lysaker et al. 2005) was applied in its German translation (MAS‐A‐G; Bröcker et al. 2017). It is an established instrument to measure metacognition, that is, the capacity “to think about thinking” in individuals with schizophreniform disorders. It comprises four subscales, namely understanding of one's mind, understanding of other's mind, decentration, and mastery. The MAS‐A‐G focuses specifically on how the ability manifests itself in free narratives of emotionally relevant situations, thereby going beyond traditional laboratory tasks. It was included as an indicator of the convergent validity of the NET. A sum score was calculated for further analyses. Sub‐scores were not considered, as the empirical preliminary analysis confirmed a one‐factor solution for the instrument (Bröcker et al. 2023).

The *Mini‐ICF‐Rating for Limitations of Activities and Participation in Psychological Disorders* (MINI‐ICF‐APP) was used to measure psychosocial functioning. The instrument is based on the International Classification of Functioning, Disability and Health (ICF) and was developed to assess impairments of psychosocial capacities and functioning (Linden and Baron 2005; Molodynski et al. 2013). The short observer rating instrument assesses four areas of functioning with 13 items: (1) ability to act (adherence to regulations, planning and structuring tasks, endurance, proactivity), (2) cognitive functioning (flexibility, applying expertise, capacity to judge and decide), (3) social functioning (power of self‐assertion, contacts with others, team‐work and group interaction capacity, dyadic relationships), and (4) basic functioning (self‐care, mobility). Each item is rated on a five‐point Likert scale, ranging from 0 (no impairment) to 4 (total disability), resulting in sum scores for the four sub scales and a global sum score ranging from 0 (best) to 52 (worst).

An intelligence quotient (IQ) of premorbid verbal intelligence as well as a measure of memory and learning were included as measures of non‐social cognition. The IQ was estimated with a German vocabulary test, the *Wortschatztest* (WST; Schmidt and Metzler 1992). Performance in memory and learning was represented by the mean score of the first five presentations of the Auditory Verbal Learning Test (AVLT; Heubrock 1992). All patients completed both tests at the baseline measurement of the MPP‐S study.

Psychopathology was assessed with three rating scales. Positive symptoms were assessed with the *Scale for the Assessment of Positive Symptoms* (SAPS; Andreasen 1984) and negative symptoms with the *Scale for the Assessment of Negative Symptoms* (SANS; Andreasen 1989). The SAPS consists of 34 items across four categories: hallucinations, delusions, bizarre/disorganized behavior, and positive formal thought disorder. The SANS includes 25 items in five categories: affective blunting, alogia, avolition‐apathy, anhedonia‐asociality, and inattention. Items are scored on a scale from 0 (none) to 5 (severe), resulting in a SAPS total score between 0 and 170 and a SANS total score between 0 and 125. Depressive symptoms were measured with the German version of the *Calgary Depression Scale for Schizophrenia* (CDSS‐G; Müller et al. 1999) which contains nine items which are rated from 0 (none) to 3 (severe). Total scores were used for further calculations.

### 2.3 Procedure

The NET interviews were conducted as part of the regular assessments at T_1_ (6 months follow‐up after baseline) of the MPP‐S study. One assessment lasted about 2 h. The NET interviews, which were included in this study, lasted on average 7.45 min ( ± 3.45 min; range: 2.36–17.54 min). Sociodemographic data were collected and a diagnostic interview, including the assessment of psychosocial functioning and symptoms, was performed.

### 2.4 Statistical Analyses

First, data was analyzed with regard to potential gender differences in variables, comparing means and frequencies using *t*‐test for independent samples, *Χ*²‐tests, or Fisher's exact test, depending on the level of measurement.

Next, Pearson correlation coefficients were calculated to examine the relationships between social cognition, metacognition and the level of social functioning.

Finally, mediation analyses were conducted using the PROCESS macro by (Hayes 2023; Preacher and Hayes 2008). Bias‐corrected and accelerated bootstrap confidence intervals with 5000 samples (BCa CI) served to calculate the indirect effect. We accepted the indirect effect as significant if its bias corrected 95% CI excluded zero. The mediation analysis was controlled for potential covariates (age, non‐social cognition, and psychopathology).

The significance level for all analyses was set at *p* < 0.05 (one‐ or two‐tailed, depending on the hypothesis). Correction of type I error in multiple tests was performed according to Bonferroni (Shaffer 1995).

Statistical analysis was performed using IBM SPSS Statistics for Windows (version 29.0, SPSS Inc., Armonk, NY, USA).

## 3 Results

### 3.1 Socio‐Demographic and Clinical Variables

There were no significant differences between men and women in terms of sociodemographic variables, premorbid verbal IQ, characteristics of illness or depressive and negative symptoms. Performance in memory and learning at baseline was significantly lower for male as compared to female patients, (*t*(90) = −2.18, two‐tailed *p* = 0.032, *d* = −0.46). Moreover, men showed significantly more positive symptoms than women, (*t*(93) = 2.20, two‐tailed *p* = 0.030, *d* = 0.47), and their NET interviews lasted significantly longer than those of female patients (*t*(93) = 2.08, two‐tailed *p* = 0.040, *d* = 0.44). For a detailed presentation of results, see Table 1.

### 3.2 Gender, Social Cognition and Psychosocial Functioning

Female patients scored significantly higher than male patients regarding psychosocial functioning (*t*(93) = 2.03, one‐tailed *p* = 0.023, *d* = 0.43), measured with the MINI‐ICF, and regarding the emotion perception index (*t*(93) = −2.25, one‐tailed *p* = 0.014, *d* = −0.47), measured with the NET. Other gender differences were not significant. For a detailed presentation of results, see Table 2. Psychosocial functioning and social cognition were significantly correlated (*r*(93) = −0.34, *p* < 0.001). Correlations of NET subscales as indicators of social cognition, MAS‐A metacognition, MINI‐ICF functioning, and symptoms (SANS, SAPS) were almost all significant and are displayed in Table 3. After Bonferroni correction for multiple testing, correlations of the NET total score and other variables remained significant, except for the SAPS. Correlations of NET subscales were less consistently correlated after Bonferroni correction but remained highly correlated with MAS‐A metacognition.

**Table 2:** Gender differences in psychosocial functioning and social cognition.

	Female		Male		*t* a	*p*	Cohen's *d*
	*M*	SD	*M*	SD			
Mini‐ICF sum score	14.43	7.76	18.09	9.03	2.03	0.023*	0.427
NET Scores							
Total NET	57.11	7.87	54.50	10.79	−1.27	0.104	−0.267
Emotion perception	16.30	2.55	15.00	2.87	−2.25	0.014*	−0.472
Theory of Mind	12.03	3.85	12.36	3.85	0.414	0.340	0.087
Attribution style	7.92	1.77	7.47	2.05	−1.11	0.135	−0.233

*Note:* Gender differences were more pronounced for individuals with schizo‐affective disorder than for those with schizophrenia.

Abbreviations: Mini‐ICF‐APP, Mini‐ICF‐Rating for Limitations of Activities and Participation in Psychological Disorders; NET, Narrative of Emotion Task.

^a^ *t*‐test for independent samples.

* one‐tailed *p* < 0.05.

**Table 3:** Inter‐correlations between measurements.

	MINI‐ICF	MAS‐A	SAPS	SANS
NET Scores				
Total NET	−0.336**,††	0.408**,††	−2.69*	−0.391**,††
Emotion perception	−0.372**,††	0.314**,††	−0.252*	−0.320**,††
Theory of Mind	−0.243*	0.320**,††	−0.084	−0.389**,††
Attribution style	−0.214*	0.360**,††	−0.245*	−0.243*

*Note:* NET = Narrative of Emotion Task; Mini‐ICF = Mini‐ICF‐Rating for Limitations of Activities and Participation in Psychological Disorders; MAS‐A = Metacognition Assessment Scale – Abbreviated; SAPS and SANS = Scale for the Assessment of Positive and Negative Symptoms.

* two‐tailed *p* < 0.05.

** two‐tailed *p* < 0.001.

^††^ Corrected *p*‐value (Bonferroni) *p* < 0.003.

### 3.3 Mediation Analysis

Mediation analysis showed no significant indirect effect of gender via social cognition measured with the NET on psychosocial functioning measured with the MINI‐ICF (both sum scores) (*b* = −0.73, 95% BCa CI [−2.18; 0.29]). This result did not change when the mediation analysis was controlled for potential covariates (age, non‐social cognition, and psychopathology) and when the mediation analysis was repeated for subscales of the MINI‐ICF. When the mediation analysis was repeated for NET subscales, results indicated that the NET emotion perception index could be considered as a mediator for gender on psychosocial functioning (*b* = −1.38, 95% BCa CI [−3.06; −0.14]). When the analysis was controlled for the above‐mentioned covariates, the significant indirect effect of NET emotion perception disappeared (see Table 4).

**Table 4:** Results of mediation analysis: Gender (X) on functioning (Y) via the mediator NET emotion processing (M).

	NET EP (M)			MINI‐ICF sum score (Y)		
	*B*	*SE*	*p*	*B*	*SE*	*p*
Gender (X)	0.383	0.691	0.581	−1.754	1.14	0.129
NET EP (M)	—	—	—	−0.396	0.193	0.044*
Covariates						
Age	0.013	0.031	0.67	0.007	0.052	0.898
Verbal IQ	−0.025	0.031	0.427	−0.046	0.052	0.382
AVLT 1‐5	0.018	0.141	0.901	0.037	0.233	0.876
SAPS sum score	−0.023	0.022	0.288	0.195	0.036	< 0.0001**
SANS sum score	−0.036	0.023	0.124	0.214	0.0.39	< 0.0001**
CDSS‐G sum score	0.036	0.08	0.654	0.561	0.561	0.0001**
	*R²* = 0.096			*R²* = 0.76		
	*F*(7, 73) = 1.11, *p* = 0.366			*F*(8, 72) = 28.569, *p* < 0.0001**		

*Note*: NET EP = Emotion perception index of the Narrative of Emotion Task; Mini‐ICF = Mini‐ICF‐Rating for Limitations of Activities and Participation in Psychological Disorders; SAPS and SANS = Scale for the Assessment of Positive and Negative Symptoms; CDSS‐G = German Calgary Depression Scale for Schizophrenia.

* two‐tailed *p* < 0.05.

** two‐tailed *p* < 0.001.

## 4 Discussion

The present study examined the relationship between gender, social cognition, and psychosocial functioning in patients with non‐affective psychosis (NAP). Social cognition was measured in an ecologically valid, narrative experimental design with the NET (Buck 2013). Consistent with Hypotheses (1) and (2) and previous research, women were rated as having a higher psychosocial functioning than men (Giordano et al. 2021; Zorrilla et al. 2015; Bucci et al. 2023), and psychosocial functioning was significantly positively correlated with social cognition (Fett et al. 2011; Halverson et al. 2019). However, results are less conclusive concerning Hypothesis (3) and (4), which claimed that female patients would show higher ratings of social cognition than male patients and that social cognition would explain part of the pathway from gender to functioning (i.e., have a mediating effect). The present study did not find gender differences in social cognition, measured with the total score of the NET, which is consistent with existing meta‐analyses (Kohler et al. 2010; Savla et al. 2013). The emotion perception index was the only subscale of the NET for which gender differences were found, meaning that female patients were scored higher for defining four emotions (fear, happiness, sadness, anger) and for choosing example situations in which they had felt the emotions. The same applied to the mediation analyses: While we did not find a mediating effect of NET (total score) for the relationship between gender and functioning, as assumed in Hypothesis (4), there was evidence for a potentially mediating role of the emotion perception index of the NET for the relationship between gender and functioning.

The results of Hypothesis (3) and (4) combined indicate that gender differences in patients with NAP may only be found for certain aspects of social cognition. NET emotion perception comprises both an individual's ability to define or identify emotions and their ability to describe and contextualize them. It differs from simple emotion perception as measured in face perception tasks, where previous research did not find significant gender differences (Navarra‐Ventura et al. 2018). One could speculate that this means that gender differences in NAP are only present for more complex social cognitive skills, especially regarding emotions. A potential explanation for this could be that women are more likely to be socialized to pay attention to, reflect on, and report on emotions. This could translate to heightened sensitivity to social and emotional cues in their surroundings, leading to superior performance on tasks requiring emotional identification and contextualization. This aligns with existing research demonstrating higher levels of empathy in healthy women (Rochat 2023). Abu‐Akel and Bo (2013) propose that female patients with NAP may exhibit better social cognitive skills before psychosis onset which could be linked to a general decreased vulnerability of developing NAP in women.

However, when we included covariates such as psychopathology and non‐social cognition in the mediation analysis, the indirect effect of gender on functioning via emotion perception was no longer significant. Thus, the indirect effect might have been confounded by gender differences in psychopathology and non‐social cognition: Male patients in this sample showed more positive symptoms and lower scores in memory and learning than female patients, pointing to their substantial role in explaining gender differences in functioning among patients. Another possibility is that results could not be maintained due to statistical limitations. While emotion perception might play a mediating role, the analysis might fail to detect the effect due to insufficient power. Taken together, the results indicate that the relationship between gender and functioning in NAP is complex and influenced by multiple interacting factors. NET social cognition (specifically emotion perception) alone does not sufficiently account for higher functioning in female patients.

The aim of Hypothesis (5) was to explore the relationship between the NET and MAS‐A‐G, and to thereby strengthen the nomological network (Cronbach and Meehl 1955) of the NET. First, the NET items showed a high internal consistency for the NET total score, but low internal consistency for sub‐scales. The internal consistency values are consistent with those in the original study by Buck et al. (2014) and results should be relativized to this. Concerning the MAS‐A‐G, subdimensions were not examined, as previous analyses indicated a single‐factor solution of the MAS‐A‐G (Bröcker et al. 2023). As expected, highly significant positive correlations were found between the NET and the MAS‐A‐G sum scores in our sample, indicating convergent validity (as shown in Table 3). Narrative approaches like the NET and MAS‐A‐G go beyond classic laboratory settings and possibly capture distinct aspects of social cognition. Of note, these results are preliminary and cannot be generalized.

### 4.1 Limitations

The most important limitation in the comparison of existing findings seems to be the heterogeneity of the instruments used and the broad definition of social cognition. Therefore, we are endeavoring to introduce an ecologically valid instrument and also to locate it empirically in the context of existing instruments. All results for the German version of the NET have to be regarded as preliminary. Similar to the original version of the NET, internal consistency was good for the total NET score but low for the social indices and the sociality subscale. Therefore, subscales should be used with caution and a psychometrical optimization of the scale should be considered. Furthermore, as this is a cross‐sectional study, no conclusions can be drawn about the direction of effect in the interaction of social cognition, psychopathological symptoms, and psychosocial functioning.

### 4.2 Implications and Conclusion

As expected, female patients with schizophrenia or schizo‐affective disorder showed higher psychosocial functioning than male patients and functioning and social cognition showed significant positive correlations. We could not find a consistent mediating effect of social cognition on the relationship between gender and functioning, most likely due to the more significant impact of psychopathology and non‐social cognition.

## Author Contributions

The second author conducted the initial literature search and wrote the first draft under supervision of the first and last author. The first and last author revised the first draft, finalized and tested the hypotheses and conducted the statistical analysis. The third and fourth author developed the idea for the study and were both responsible for and involved in its conduct. All authors critically read and revised the manuscript.

## Conflicts of Interest

The authors declare that the research was conducted in the absence of any commercial or financial relationships that could be construed as a potential conflict of interest.

## Supporting information

NET AppendixA.

## Data Availability

The data that support the findings of this study are available from the corresponding author upon reasonable request. The data are not publicly available due to privacy or ethical restrictions.

## Endnotes

1 It should be noted that gender is a fluid and multifaceted concept (see e.g., APA, 2015). Since the existing studies in schizophrenia research employ a binary distinction (female vs. male), we follow this binary distinction in this study to ensure comparability.
