# Supplementary material for: Gender Differences in Social Cognition and Their Association With Functioning in Individuals With Non‐Affective Psychosis
Source: J Clin Psychol. 2025 Oct 14;82(2):161–8. doi: 10.1002/jclp.70052 (PMC12793826; doi:10.1002/jclp.70052)
Supplement: Supplementary file 1 — NET AppendixA. [file JCLP-82-161-s001.docx]

**Appendix A**

**Task Structure of the Narrative of Emotion Task**

(adapted from Buck, 2013)

1. Was bedeutet _______ für Sie? (**Definition**)
   *English original: What does ________ mean?*
2. Schildern Sie eine Situation, in der Sie _______ empfunden haben. (**Memory**)
   *English original: Can you tell me about a time when you felt _______?*
3. Warum haben Sie in dieser Situation _______ empfunden? (**Prompt**)
   *English original: Why did that make you feel _______?*

Emotions:

1. Traurigkeit *(Sad)*
2. Angst *(Afraid)*
3. Wut *(Angry)*
4. Freude *(Happy)*

**Appendix B**

**Coding Manual for the Narrative of Emotion Task**

(adapted from Buck, 2013)

**Definition of Emotion**

0 = no response or “I don’t know”

1 = attempted but incorrect response; response is a related emotion or an example of a related emotion; response contains the emotion as an integral part of the definition

2 = response was an appropriate example but not a definition

3 = correct definition

**Presence of Narrative**

0 = no response, “I don’t know”, OR fewer than two clauses

1 = clauses do not cast participant as the protagonist in a specific narrative account

2 = clauses do not connect temporally (no time passes)

3 = two or more clauses, story is a specific narrative account (versus a general statement), story is told from a first-person evaluative perspective where the participant is cast as protagonist, and clauses are connected by some temporal arrangement (where time passes)

**Contextually Appropriate Circumstances**

0 = no response or “I don’t know”

1 = incorrect/inappropriate context

2 = moderately appropriate context/response is a rewording of example given by interviewer

3 = appropriate context

**Causal Inferences**

0 = no response or “I don’t know”

1 = no causal circumstances given, or causal circumstances do not connect event to

emotion

2 = causal circumstances included, but only with PROMPT

3 = causal circumstances included within the MEMORY (without PROMPT)

**Clarity of Meaning**

0 = no response or “I don’t know”

1 = unclear; significant difficulty in making sense of the participant’s response OR 3

or more of the examples noted above

2 = moderately clear; general meaning of response is relatively preserved but some disorganization detracts from it being wholly clear OR 1-2 of examples noted above

3 = clear; response is clear and organized

**Clarity of Grammar**

0 = no response or “I don’t know”

1 = unclear; grammatical errors greatly impair sentence structure and/or the ability to

comprehend the response (usually around 3+ grammatical errors)

2 = moderately clear; some grammatical errors but they do not largely detract from the structural clarity of speech segment (usually 1-2 grammatical errors)

3 = clear; speech segment is free of grammatical errors

**Elaboration**

0 = no response or “I don’t know”

1 = not elaborated; 0 additional pieces of information

2 = moderately elaborated; 1-2 additional pieces of information

3 = very well elaborated; 3+ additional pieces of information
